# Duck TRIM32 Functions in IFN-β Signaling Against the Infection of H5N6 Highly Pathogenic Avian Influenza Virus

**DOI:** 10.3389/fimmu.2020.00377

**Published:** 2020-02-28

**Authors:** Siyu Wu, Junsheng Zhang, Qian Xue, Jing Liu, Bingzhong Huang, Zhuoliang He, Jianni Huang, Shaopo Zu, Zuxian Chen, Bingbing Zhao, Ming Liao, Peirong Jiao

**Affiliations:** ^1^College of Veterinary Medicine, South China Agricultural University, Guangzhou, China; ^2^Guangdong Laboratory for Lingnan Modern Agriculture, Guangzhou, China

**Keywords:** duck, TRIM32, STING, IFN-β, avian influenza virus

## Abstract

In mammals, tripartite motif 32 (TRIM32) is essential for regulating host innate immune responses to viral infections. However, the antiviral effect of TRIM32 in birds has not been reported. Here, we cloned the full-length duck TRIM32 (duTRIM32) cDNA from total spleen RNA of Peking duck. DuTRIM32 consists of 682 amino acids and has 95.5% similarity in amino acid sequences with chicken TRIM32 and 84.9% similarity with human TRIM32, respectively. DuTRIM32 mRNA was found to be ubiquitously expressed in all tested tissues from healthy ducks. Overexpression of duTRIM32 significantly activated the IFN-β promoter and upregulated the mRNA levels of IFN-β, IRF7, and Mx, which indicates that duTRIM32 is involved in the type I IFN pathway. Furthermore, duTRIM32 was found to directly interact with duck STING (duSTING) and to contribute to the expression of IFN-β mediated by duSTING. The mRNA level of duTRIM32 was significantly upregulated in the lungs and spleens of H5N6 highly pathogenic avian influenza virus (HPAIV) infected ducks 3 days post-infection (DPI). Furthermore, overexpression of duTRIM32 could inhibit the replication of H5N6 HPAIV in duck embryo fibroblasts (DEFs). Therefore, these results indicate that duTRIM32 is involved in the type I IFN pathway and exhibit an antiviral effect against H5N6 HPAIV infection.

## Introduction

After viral infection, the host innate immune system recognizes the conserved viral components named pathogen-associated molecular patterns (PAMPs) by host pattern recognition receptors (PRRs) (1). The recognition of PAMPs by PRRs activates the innate immune signaling pathway that induces the expression of type I interferons (IFNs) and pro-inflammatory cytokines to control viral infection (2). Various post-translational modifications such as phosphorylation, ubiquitination, methylation, and acetylation have emerged as critical regulators of immunity by influencing the PRR-dependent pathway (3). Protein ubiquitination is one of the major conventional post-translational modifications that regulate the innate immune signaling pathway by targeting PRRs or downstream adaptor molecules (4)_._ The ubiquitination process is a unique enzymatic cascade reaction. First, ubiquitin-activating enzymes (E1) activates ubiquitin in an ATP-dependent manner and then the activated ubiquitin is transferred to E2 ubiquitin-conjugating enzymes. Finally, E3 ubiquitin-ligating enzymes facilitate ubiquitination of the substrate. E3 ubiquitin-ligating enzymes are the center of this process that determines the specificity of the substrate (5).

TRIM proteins are a family of E3 ubiquitin-ligating enzymes that contain a conserved N-terminal RBCC domain (RING finger/B-box/coiled coil) (6). Mammalian TRIM proteins could modulate the innate immune response by ubiquitinating the key molecules of the signaling pathway. For example, TRIM56 could catalyze STING for K63-linked ubiquitination to induce STING dimerization, thereby enhancing the innate immune response (7). TRIM31 could also interact with mitochondrial antiviral-signaling protein (MAVS) and target it to K63-linked polyubiquitination to promote the RIG-I-MAVS-mediated signaling pathways (8). In addition, some of the TRIM proteins have been demonstrated to inhibit viral replication by directly targeting viral proteins. For example, both TRIM14 and TRIM22 have been reported to inhibit the replication of HCV by targeting NS5A protein for degradation (9, 10). Recently, mammalian TRIM32 has attracted much attention for its critical roles in enhancing viral-triggered innate immune responses (11). TRIM32 could directly interact with PB1 to inhibit the replication of the influenza A virus (12). However, the role of TRIM32 in the bird innate immune signaling pathway has not been reported.

In mammals, the cyclic GMP-AMP synthase (cGAS) may sense and react to DNA viral infections via the cGAS-STING pathway (13). RIG-I like receptors could sense RNA virus infection to trigger an innate immune response through the RIG-I-MAVS pathway (14, 15). STING is reported to participate in the RIG-I-MAVS pathways and is crucial for anti-RNA viral infection (16–18). Several TRIM proteins, including TRIM29, TRIM30α, TRIM32, and TRIM56 have been reported to regulate the mammalian STING-mediated pathways (11, 19, 20). DuSTING was reported to activate the type I IFN pathway and exhibited antiviral effects against both DNA and RNA viruses (21, 22). However, whether duTRIM32 regulates the duSTING-mediated pathway remains unknown.

In this study, we cloned the TRIM32 coding sequence from Peking duck and investigated the role of duTRIM32 in the type I IFN pathway and its antiviral effect on H5N6 HPAIV infection.

## Materials and Methods

### Viruses, Cells, and Animals

The H5N6 virus A/duck/Guangdong/16873/2016 (DK16873) used in this study was isolated from swabs of ducks at a live bird market in Guangdong, China in 2016. The H5N6 virus was isolated in 9-day-old specific-pathogen-free (SPF) embryonated hen eggs, using methods described in the literature (23). The 50% tissue culture infective dose (TCID_50_) of the H5N6 virus was determined by infection of DEFs; 50% egg infective doses (EID_50_) was determined by infection of embryonated hen eggs, and values were calculated using the Reed-Muench method (24). DEFs were derived from 9-day-old duck embryos and cultured in Dulbecco's modified Eagle medium (GIBCO, USA) complemented with 10% fetal bovine serum (GIBCO, USA). Four-week-old healthy Peking ducks were purchased from farms in Guangdong and housed in the isolators of the ABSL-3 facilities. The Peking ducks were confirmed to be serologically negative for avian influenza with a hemagglutination inhibition (HI) test.

### Cloning and Sequence Analysis of the duTRIM32

According to the predicted sequence of duck-TRIM32 (duTRIM32) from the National Center for Biotechnology Information (Accession number: XP_005029948.1), primers were designed and used to amplify full-length coding sequence (CDS) of duTRIM32 cDNA from total spleen RNA of Peking ducks (Table 1). Amino acid sequences of different species TRIM32 were aligned using Clustal X2.1 software and edited with BOXSHADE (https://embnet.vital-it.ch/software/BOX_form.html). The functional domains of duTRIM32 were predicted using the SMART program. MEGA software (vesion 4.0) was also used to conduct phylogenetic analysis of TRIM32 amino acid sequences of 10 species.

**Table 1:** Primer sequences used in this study.

**Gene**	**Primer sequences (5^′^ to 3^′^)**	**Purpose for**
duTRIM32-F	ATGGCAACCACGGCACTGAA	To obtain sequence
duTRIM32-R	TTAAGGGGTGGAATATCT	
duTRIM32-V5-F	TATGAATTCATGGCAACCACGGCACTGAA	Gene cloning
duTRIM32-V5-R	TCGCTCGAGTTACGTAGAATCGAGACCGAGGAGAGGGTTAGGGATAGGCTTACCAGGGGTGGAATATCT	
duTRIM32-q-F	TCGCTGTGATTATTTCCTCG	qRT-PCR
duTRIM32-q-R	TCACCACCTGCCCGATCTGC	
duIFN-β-q-F	CAGCATCAACAAGTACTTCA	qRT-PCR
duIFN-β-q-R	CTTCCGAAGTGGCTGGGAGA	
duIRF7-q-F	CCACACCTGGATGTCACCAT	qRT-PCR
duIRF7-q-R	AGACGTGCTGCCCCGGCTGC	
duMx-q-F	CCAGACCTGACACTAATTGAT	qRT-PCR
duMx-q-R	CACATTACATGGCACCACTAC	
GAPDH-q-F	ATGTTCGTGATGGGTGTGAA	qRT-PCR
GAPDH-q-R	CTGTCTTCGTGTGTGGCTGT	

### Expression Profiles of duTRIM32 in Healthy Ducks and H5N6 HPAIV Infected Ducks

To analyze the distribution of duTRIM32 in healthy ducks, three 4-week-old healthy ducks were euthanized humanely to collect tissues that included livers, pancreases, hearts, brains, kidneys, lungs, spleens, intestines, thymuses, bursa of Fabricius, and stomachs. The lung, spleen and brains are the major target tissues for H5 subtype AIVs infection, and our previous study have demonstrated that the host immune responses in these organs may influence the outcome of the infection (25). Therefore, we have quantified the expression of duTRIM32 in the lungs, spleens, and brains of the infected ducks to evaluate if duTRIM32 involved in the host immune response to H5N6 infection. To investigate the expression of duTRIM32 in H5N6 infected ducks, six 4-week-old ducks were inoculated intranasally with 10^6^ EID_50_ of DK16873 H5N6 HPAIV in a volume of 200 uL. At 12 h and 3 days post-inoculation, each of the three inoculated birds were euthanized humanely to collect lungs, brains and spleens. Three control ducks were treated with PBS in the same way and were also euthanized humanely to collect lungs, brains and spleens. Total RNA of the collected tissues were extracted using an Eastep®Super Total RNA Extraction Kit (Promega, USA) according to the manufacturer's instructions. Total RNA (1 μg) was reverse-transcribed into cDNA using M-MLV Reverse Transcriptase (Promega, USA) according to the manufacturer's protocol. Quantitative real-time PCR (qRT-PCR) was conducted using a GoTaq® qPCR Master Mix (Promega, USA) and was run on a Bio-Rad CFX96 Touch™ Real-Time PCR Detection System (Bio-Rad, USA) with the following settings: 5 min at 95°C, then 15 s at 95°C and 34 s at 62°C for 40 cycles.

### Luciferase Reporter Assay

The obtained sequence of full length duTRIM32 was subcloned into the pCAGGS plasmid with specific primers (Table 1). Other plasmids and luciferase reporter plasmids used in this study (duSTING-HA, pGL3-IFN-β- Luc, pRL-TK, and pCAGGS-vector) were kept in our labatory and were constructed using previously described methods (26). For the luciferase reporter assay, expression plasimids (duTRIM32-V5 and duSTING-HA), reporter plasmids, pCAGGS-vector and pRL-TK were transfected into DEFs using the FuGENE HD transfection reagent (Promega, USA). The pRL-TK plasmid (Promega, Madison, WI, USA), encoding the Renilla luciferase protein, was used as an internal control plasmid to normalize transfection efficiency. In addition, we also used pAc-GFP to observe the transfection efficiency. After 24 h, DEFs were collected and tested for luciferase activities using the dual-luciferase Assay System (Promega, USA) according to the manufacturer's instructions.

### Overexpression of duTRIM32 and qRT-PCR Analysis

DEFs were seeded on 6-well plates and cultured until the cells were 70% confluent. DuTRIM32-V5 or pCAGGS-vector was then transfected into the corresponding well (*n* = 3) using the FuGENE HD transfection reagent (Promega, USA) and cultured at 37°C with 5% CO_2_. After 24 h, DEFs were collected and the mRNA of IFN-β, IRF7, and Mx were tested using qRT-PCR described above.

### Coimmunoprecipitation Analysis

Coimmunoprecipitation (Co-IP) assays were conducted to analyze the interaction between duTRIM32 and duSTING. For Co-IP experiments, 293T cells were seeded on 100 mm dishes (10^7^ cells/dish) overnight. Expression plasmids or empty plasmids were used to transfect 293T cells. After 24 h, cells were lysed using cell lysis buffer (P0013, Beyotime, China) containing 1 mM PMSF. Lysates were centrifuged at 12,000 g, for 15 min at 4°C and precipitated with anti-HA mAb, in conjunction with protein G Agarose beads (ThermoFisher Scientific, USA) for at least 4 h at 4°C. The beads were washed with cold PBS 5 times (10 min/wash), and subsequently eluted with SDS loading buffer by boiling for 10 min. Proteins isolated from the beads and the cell lysates were separated by SDS-PAGE. In addition, the separated proteins were electroblotted onto polyvinylidene fluoride (PVDF) membranes (Millipore, Bedford, MA, USA). The membranes was blocked with 5% BSA in phosphate buffer solution and was then incubated for 1 h with mouse anti-HA and anti-V5 antibodies (Sigma, German) at a dilution of 1:2,000 and 1:3,000, respectively. The goat anti-mouse-IRDye 800 (LI-COR Biosciences, Lincoln, NE USA) was used as the secondary antibody at a 1:10,000. dilution. To determine whether ubiquitination of duSTING can be detected after the interaction, the 293T cells were co-transfected with duSTING-Myc, Ub-HA, and duTRIM32-V5. The duSTING-myc and Ub-HA transfected group was used as a control group. Cell lysates were immunoprecipitated with anti-Myc mAb, in conjunction with protein G Agarose beads (ThermoFisher Scientific, USA) and the immunoprecipitates were analyzed by Western blot with mouse anti-HA antibodies (Sigma, German) at a dilution of 1:2,000. The secondary antibody was indicated before.

### Antiviral Effect of duTRIM32 on H5N6 HPAIV

Antiviral experiments were conducted using previously described methods (26). DEFs were seeded on 12-well plates and cultured until cells were 70% confluent. Expression plasmids (duTRIM32-V5) or empty vectors was transfected into DEFs using FuGENE HD transfection reagent. After 24 h, DEFs were infected with DK16873 H5N6 virus at a dose of 5 TCID_50_. Cell supernatants were collected at 12, 24, 36, and 48 HPI. DEFs were used to determine TCID_50_ titers.

### Ethics Statement

All experiments with H5N6 HPAIV were conducted in Animal Biosafety Level 3 (ABSL-3) and Biosafety Level 3 (BSL-3) facilities. The protocol (SCAUABSL2017-011) was approved by the Experimental Animal Administration and Ethics Committee of the South China Agricultural University.

### Statistical Analysis

GraphPad Prism 7.0 software (GraphPad Software Inc, USA) was used to perform statistical analysis. The Student's *t*-test was used to analyze the statistical significance of the differences. A *p*-value < 0.05 was considered to be statistically significant (^*^p < 0.05; ^**^p < 0.01).

## Results

### Cloning and Sequence Analysis of the duTRIM32

**Figure 1:**
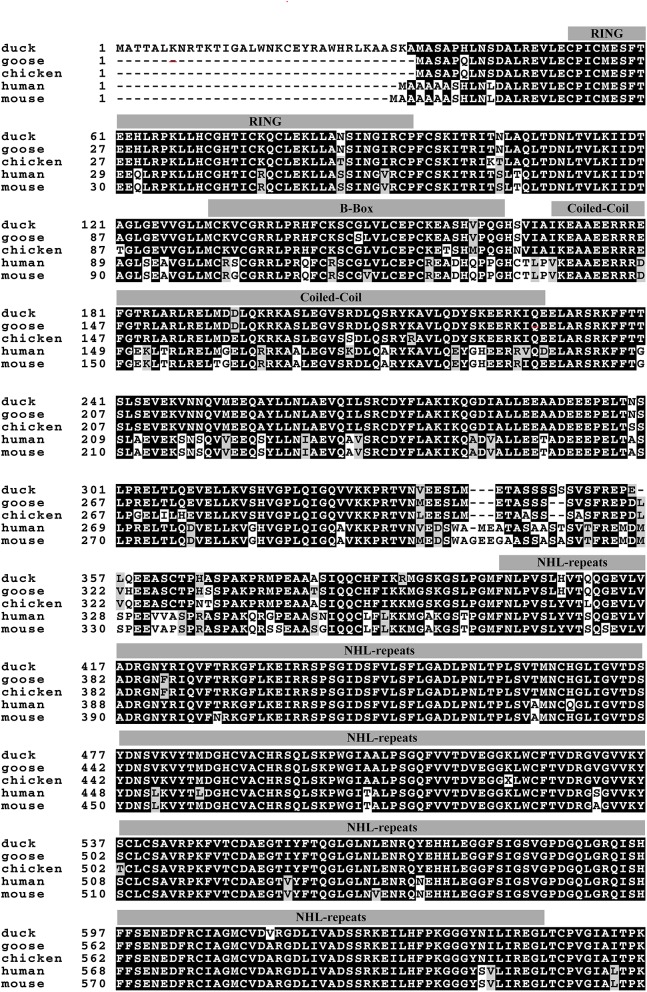
Alignment of Peking duck TRIM32 amino acid sequence with goose, chicken, human, and mouse TRIM32. The sequences of TRIM32 are derived from GenBank. The accession numbers are goose (XP_013038246.1), chicken (XP_015135179.2), human (NP_036342.2), and mouse (NP_444314.2). The predicted motifs of duck TRIM32 (RING, a B box/coiled-coil domain and a NHL domain) were indicated on the sequences.

**Figure 2:**
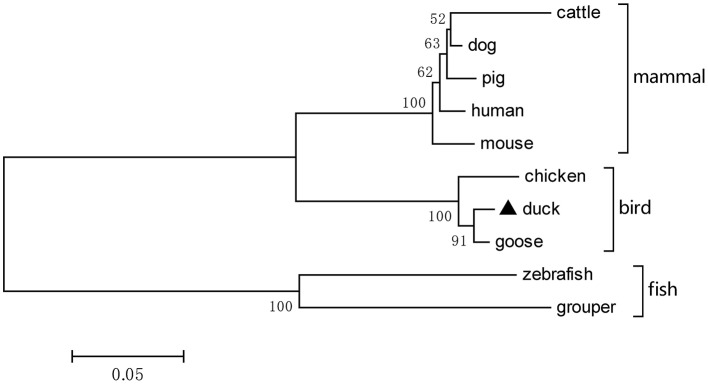
A phylogenetic tree of duck TRIM32 and other animal TRIM32 were generated by MEGA 4.0 with the neighbor-joining method. The sequences are derived from GenBank. The accession numbers are goose (XP_013038246.1), chicken (XP_015135179.2), human (NP_036342.2), mouse (NP_444314.2), cattle (NP_001069292.1), dog (XP_005627017.1), pig (XP_005660438.1), zebrafish (NP_001107066.1), and grouper (AQD20583.1).

Based on the predicted sequence of duTRIM32 on the National Center for Biotechnology Information (NCBI), primers were designed and used to amplify full-length duTRIM32 cDNA from total duck spleen RNA. The full-length duTRIM32 contains 2,049 bp, encoding 682 amino acids (Figure 1). Sequence alignment results revealed that duTRIM32 shares 98.3% similarity in amino acid sequences with goose TRIM32, 95.5% with chicken TRIM32, 84.9% with human TRIM32, 84.3 % with mouse TRIM32 and 61.5% with fish TRIM32, respectively. Phylogenetic analysis revealed that duTRIM32 is clustered into the bird clade (Figure 2). The SMART program and the well-understood structure of human TRIM32 were used to predict the functional domains of duTRIM32. Results indicate that duTRIM32 contains a conserved RING-finger domain, a BBOX domain and a coiled-coil domain, which are similar to those of mammalian TRIM32 (Figure 2).

### Expression Profiles of duTRIM32 in Healthy Ducks

**Figure 3:**
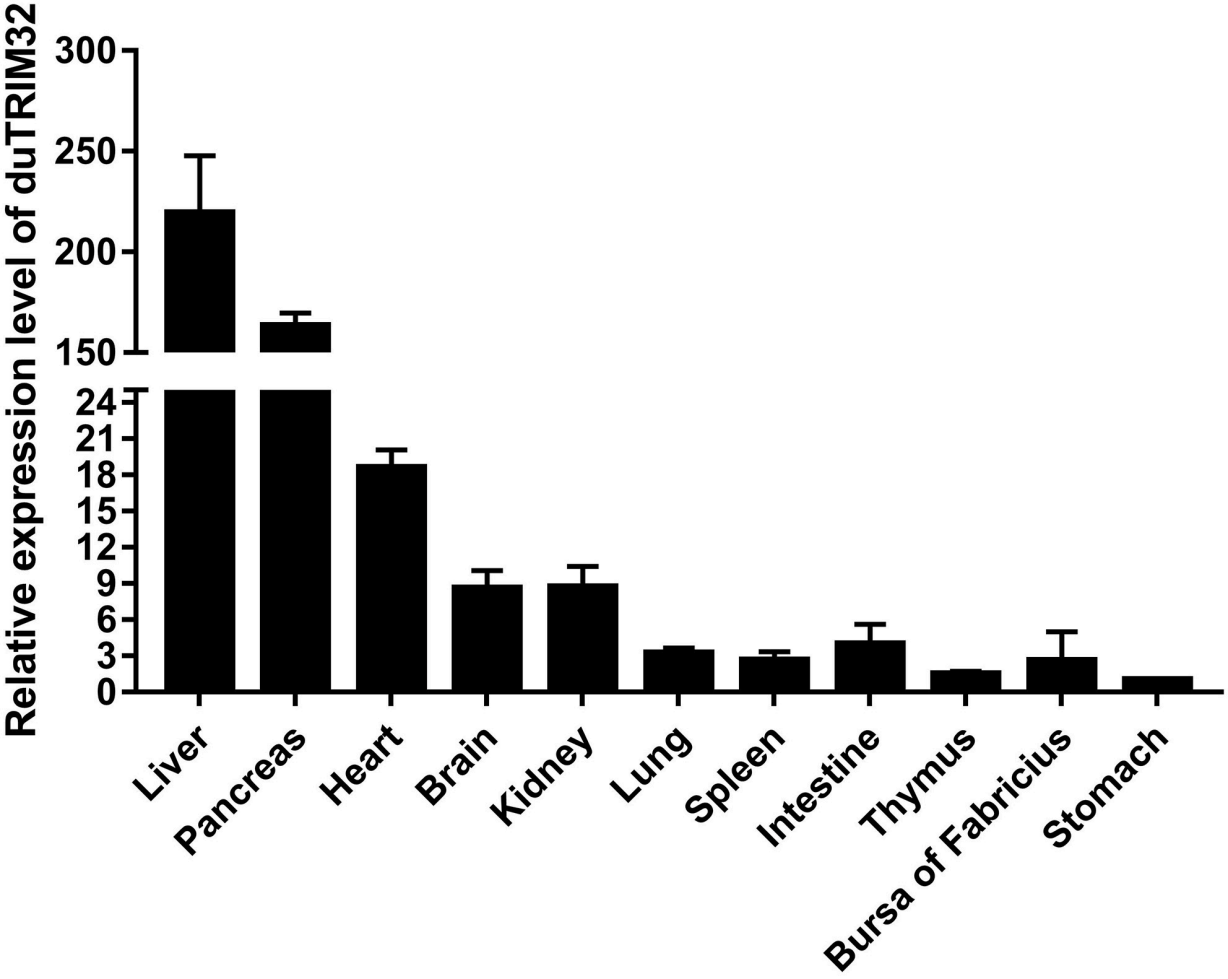
Relative expression levels of duTRIM32 in healthy ducks. The real-time RT-PCR was conducted to detect the transcription levels of duTRIM32 in ducks' tissues. GAPDH was chosen as the control gene. Data were normalized to the stomach and error bars indicated the SD.

To analyze the expression profiles of duTRIM32 in healthy ducks, we performed qRT-PCR to determine the expression of duTRIM32 in different tissues. qRT-PCR assays were conducted as described in the Materials and Methods. Results indicate the highest expression of duTRIM32 is in the liver, followed by the pancreas, heart, brain, kidney, lung, spleen, intestine, thymus, bursa of Fabricius, and stomach (Figure 3). Therefore, our results indicated that duTRIM32 is ubiquitously expressed in all the tested tissues of healthy ducks.

### Expression of duTRIM32 in Ducks Infected With H5N6 HPAIV

**Figure 4:**
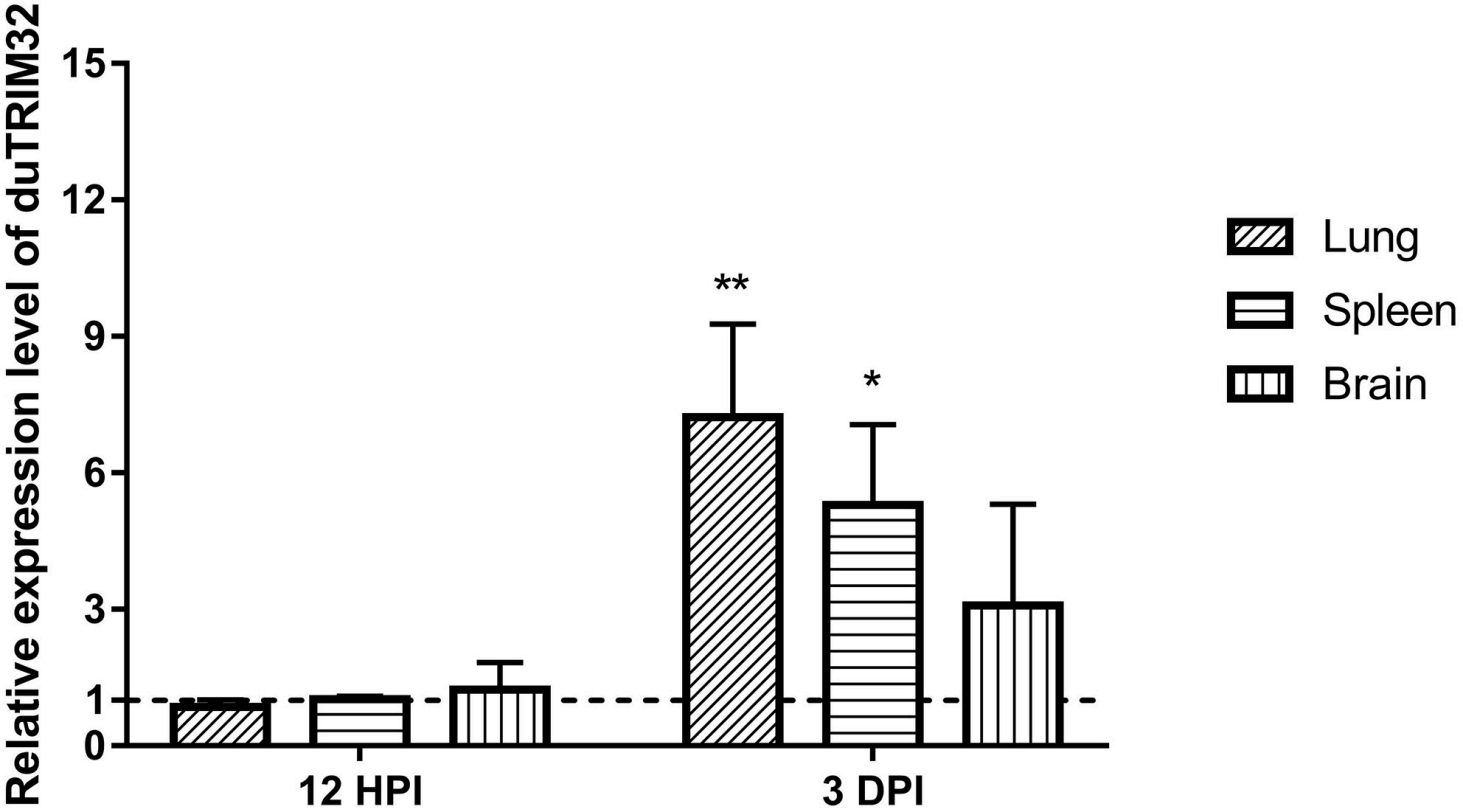
Relative expression levels of duTRIM32 in the lungs, spleens and brains of DK16873 H5N6 HPAIV infected ducks were quantified by real-time RT-PCR. The experimental ducks were inoculated with 10^6^ EID_50_ of DK16873 H5N6 HPAIVs. Control ducks were treated with PBS in the same way. Each bar shows the mRNA level of the target gene relative to those in the control group. All data were expressed as means ± SD, the Student's *t*-test was used to analyze the differences. (*p < 0.05; **p < 0.01).

To investigate if duTRIM32 is involved in the host immune response to viral infection, we quantified the mRNA levels of duTRIM32 in the lungs, spleens, and brains of H5N6 HPAIV infected ducks using techniques described in the Materials and Methods. Results from these studies indicated that the mRNA of duTRIM32 is slightly changed at 12 HPI in the lungs, spleens and brains. Notably, duTRIM32 was found to be upregulated in these tissues of the H5N6 HPAIV infected ducks at 3 days post-infection (DPI), especially in the lungs and spleens, with a fold increases of 7.23 (*P* < 0.05) and 5.29 (*P* < 0.05), respectively (Figure 4). Therefore, our results suggest that duTRIM32 might be involved in the host innate immune response to H5N6 HPAIV infection.

### Overexpression of duTRIM32 Activates the Type I IFN Pathway

**Figure 5:**
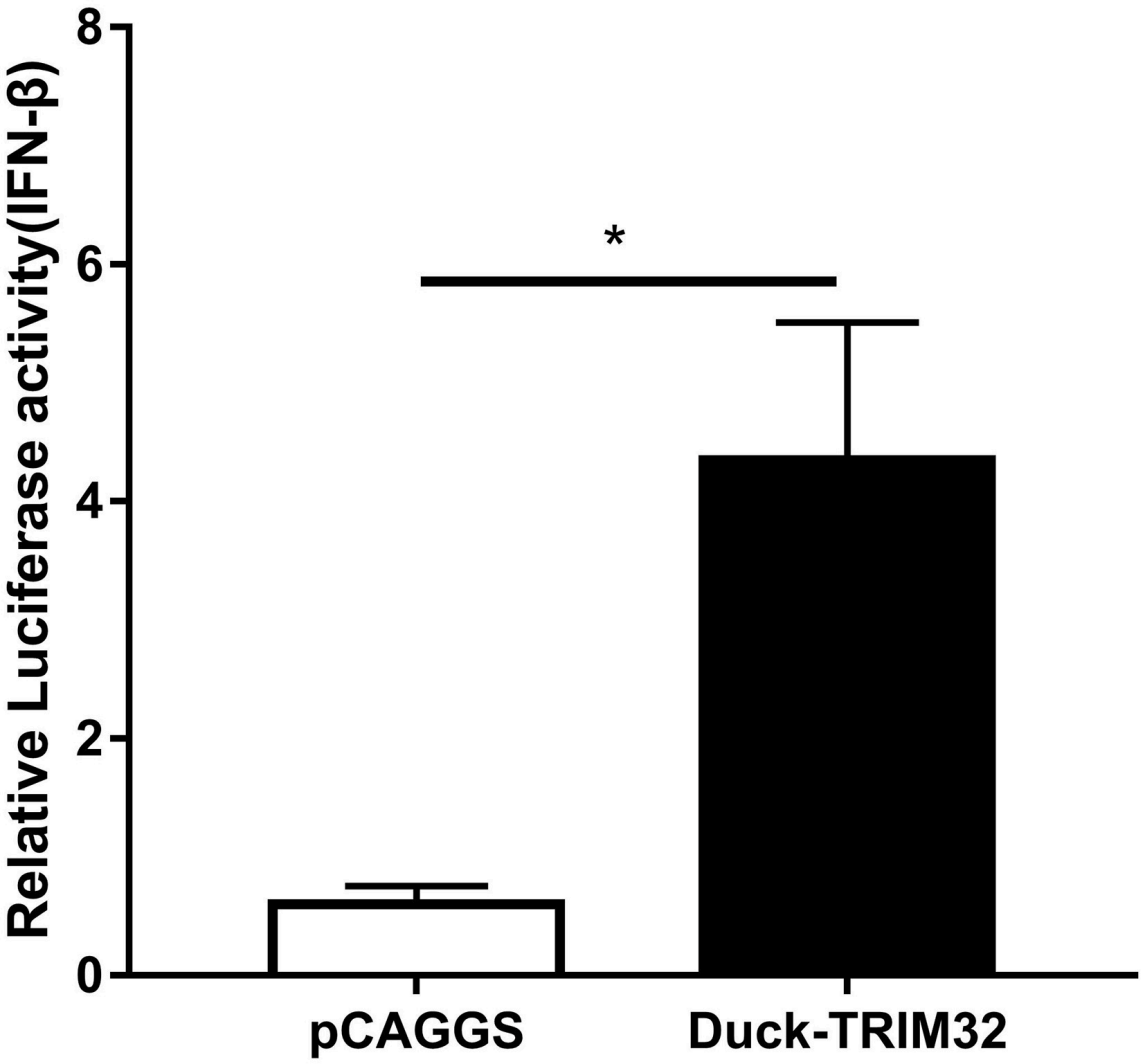
Overexpression of duTRIM32 activates the IFN-β promoter. The DEFs (24 wells) were co-transfected with pGL3-IFN-β-Luc (0.1 μg/well), PRL-TK plasmids (0.01 μg/well), pCAGGS-duSTING-HA (0.3 μg/well), or empty vector (0.3 μg/well). All data were expressed as means ± SD, the Student's *t*-test was used to analyze the differences. (*p < 0.05).

**Figure 6:**
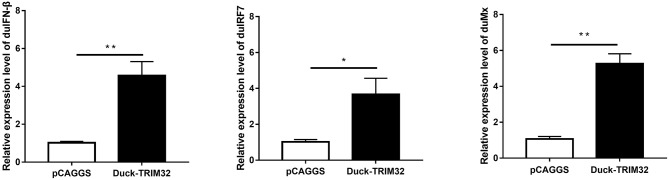
The DEFs respond to overexpression of the duTRIM32. The DEFs (6 wells) were transfected with pCAGGS-duTRIM32-V5 (2 μg/well) or empty vector (2 μg/well). The relative expression of IFNβ, IRF7, and Mx genes were detected by real-time RT-PCR. All data were expressed as means ± SD, the Student's *t*-test was used to analyze the differences. (*p < 0.05; **p < 0.01).

To determine whether duTRIM32 functions in the type I IFN pathway, we performed a luciferase reporter assay to test the ability of duTRIM32 to activate the IFN-β promoter. The luciferase reporter assay was completed as described in the Materials and Methods. The pCAGGS is an empty plasmid and used as a negative control in this study. The results show that transfection of pCAGGS could not activate the IFN-β promoter (Figure 5). These results indicate that overexpression of duTRIM32 can significantly activate the IFN-β promoter (4.34-fold, *P* < 0.05) (Figure 5). In addition, we also quantified the mRNA of IFN-β, IRF7 and Mx in the duTRIM32-transfected DEFs. Results from these studies indicate that duTRIM32 significantly upregulates the expression of IFN-β (4.58-fold, *P* < 0.05), IRF7 (3.67-fold, *P* < 0.05) and Mx (5.26-fold, *P* < 0.05) (Figure 6). Therefore, these results reveal that duTRIM32 can activate the type I IFN pathway and upregulates the expression of the related antiviral genes.

### duTRIM32 Interacts With duSTING and Contributes to the Expression of IFN-β Mediated by duSTING

**Figure 7:**
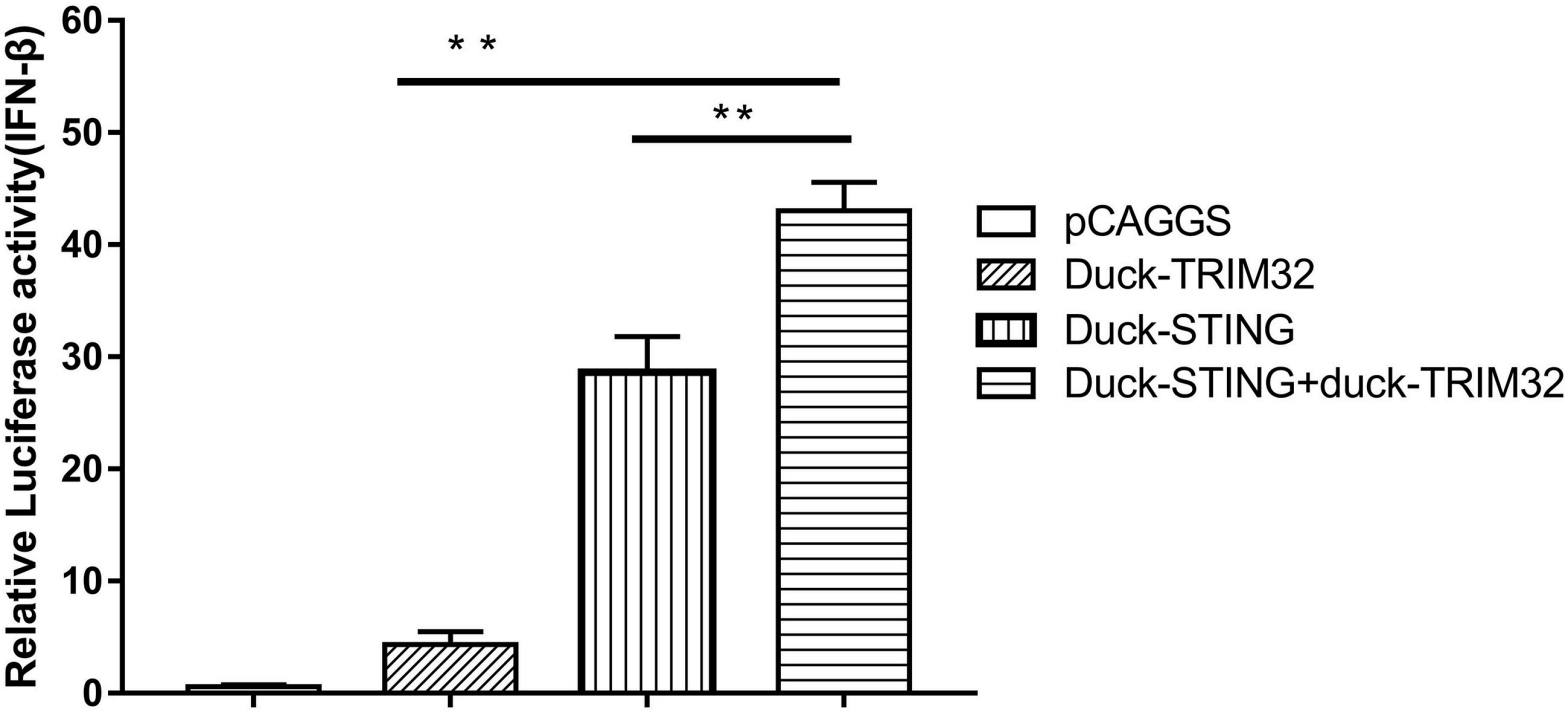
DuTRIM32 contributes to duSTING-mediated expression of IFN-β. The DEFs (24 wells) were co-transfected with pGL3-IFN-β-Luc (0.1 μg/well), PRL-TK plasmids (0.01 μg/well), pCAGGS-duTRIM32-V5 (0.3 μg/well), and/or pCAGGS-duSTING-HA (0.3 μg/well). After 24 h, DEFs were collected and tested for luciferase activities using the dual-luciferase Assay System. All data were expressed as means ± SD, the Student's *t*-test was used to analyze the differences. (**p < 0.01).

Mammalian TRIM32 was found to interact with STING and to contribute to the expression of IFN-β mediated by duSTING, thereby positively regulating the type I IFN pathway (11). We performed a dual luciferase reporter assay to determine whether duTRIM32 contributes to duSTING-mediated expression of IFN-β. Results from these studies showed that both duTRIM32 and duSTING can activate the IFN-β promoter, with fold increases of 4.34 (*P* < 0.05) and 28.63 (*P* < 0.05), respectively. Co-transfection of duTRIM32 and duSTING exhibited stronger activation of the IFN-β promoter than the transfection of duSTING, with a fold increase of 43.03 (*P* < 0.05). But the increased level between duTRIM32 and duSTING co-transfection group and duSTING transfection group is <2-folds (Figure 7).

**Figure 8:**
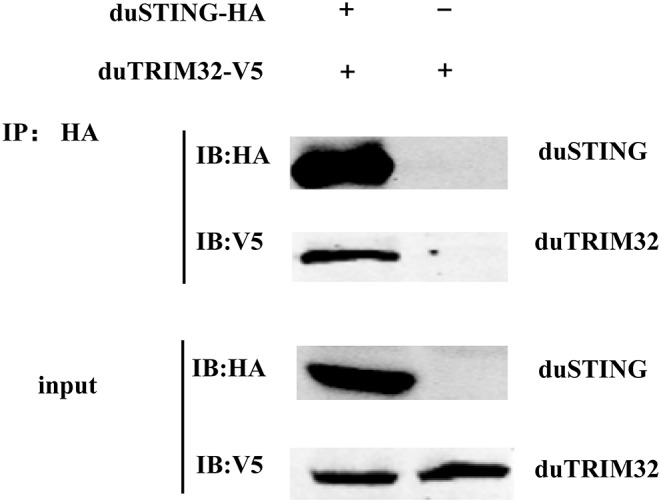
Identification of the interaction between duTRIM32 and duSTING by Co-IP assays. The 293T cells were transfected with the pCAGGS-duTRIM32-V5 and/or pCAGGS-duSTING-HA. After 24 h, the lysates were immunoprecipitated with anti-HA beads, followed by immunoblotting analysis with the indicated Abs.

**Figure 9:**
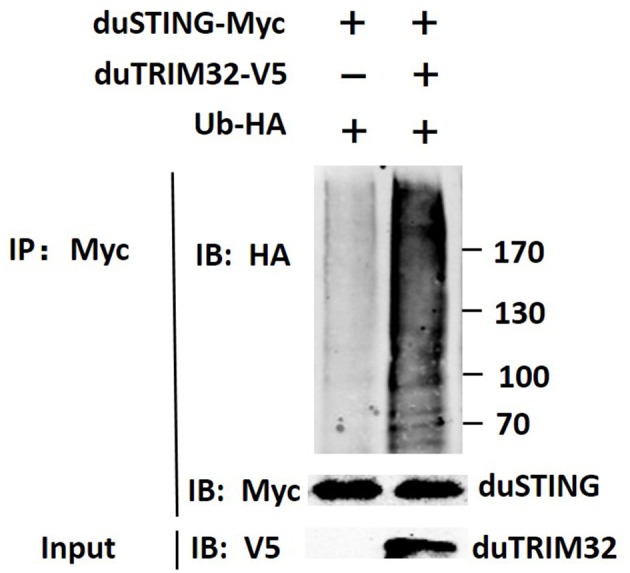
Ubiquitination of duSTING by duTRIM32. The 293T cells were co-transfected with duSTING-Myc, Ub-HA, and duTRIM32-V5. The duSTING-Myc and Ub-HA transfected group was used as a control group. Cell lysates were immunoprecipitated with anti-Myc mAb, in conjunction with protein G Agarose beads (ThermoFisher Scientific, USA) and the immunoprecipitates were analyzed by Western blot.

In addition, we co-transfected duTRIM32 and duSTING expression plasmids into 293T cells and the interaction between duTRIM32 and duSTING was analyzed by Co-IP assays. Results showed that duTRIM32 directly interacted with duSTING. Both duTRIM32 and duSTING were correctly expressed in 293T cells, with a molecular weight of 76 kDa and 43 kDa, respectively (Figure 8, Supplementary Material). These results confirmed that duTRIM32 can directly interact with duSTING. Furthermore, we found that duTRIM32 was demonstrated to ubiquitinate duSTING in 293T cells (Figure 9).

Therefore, our results revealed that duTRIM32 can interact with duSTING and contributes to duSTING-mediated expression of IFN-β.

### Antiviral Effect of duTRIM32 on H5N6 HPAIV Infection

**Figure 10:**
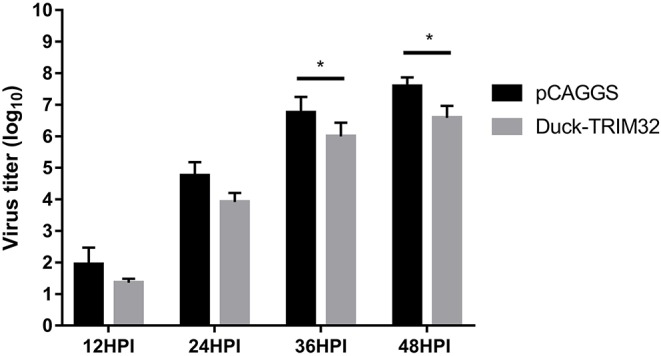
DuTRIM32 inhibits the replication of H5N6 HPAIV in DEFs. The DEFs (12 wells) were transfected with either the pCAGGS-duTRIM32-V5 (1 μg/well) or the empty vector (1 μg/well). At 24 h post-transfection, the transfected cells were infected with DK16873 H5N6 HPAIVs (5 TCID_50_). Cell supernatants were collected at 12, 24, 36, and 48 h post-infection and analyzed for their TCID_50_ titers in DEFs. Virus titers are expressed as means ± standard deviation in log_10_ TCID_50_/mL, and the Student's *t*-test was used to analyze the differences. (*p < 0.05).

To investigate the antiviral effects of duTRIM32, we transfected duTRIM32 or an empty vector in DEFs. DEFs were then infected with DK16873 H5N6 HPAIV at 24 h post-transfection. Samples were collected at 12, 24, 36, and 48 h post-infection. The viral titers of the collected samples were measured by the TCID_50_ method in DEFs. Results indicated that the viral titers of H5N6 HPAIV in the duTRIM32-transfected group were lower than that of the empty vector transfected group. Notably, overexpression of duTRIM32 could significantly inhibit the replication of H5N6 HPAIV at 24 HPI (*P* < 0.05) and 36 HPI (*P* < 0.05) (Figure 10). Therefore, our results revealed that duTRIM32 can inhibit the H5N6 HPAIV infection.

## Discussion

TRIM proteins are involved in various cellar physiological processes, such as cell differentiation, apoptosis, tumorigenesis, and innate immunity (27). Over the last decade, more and more mammalian TRIM proteins were found to be important immunomodulatory effectors of the innate immunity against viral infections. For example, TRIM5, TRIM21, TRIM23, TRIM31, TRIM32, TRIM56, and TRIM65 are reported to positively regulate the type I IFN pathway (7, 8, 11, 28–31). Some TRIM proteins, like TRIM26 and TRIM40 are reported to act as negative regulators for innate immune responses (32, 33). Among them, mammalian TRIM32 have been well-elucidated for its critical roles in regulating antiviral innate immune responses (11). However, the antiviral effect of TRIM32 in birds has not been reported.

In our study, we cloned the full-length duTRIM32 cDNA from Peking duck. DuTRIM32 was ubiquitously expressed in all the tested tissues of healthy ducks, which suggests that duTRIM32 might be involved in various cellar processes. Phylogenetic analysis revealed that duTRIM32 is clustered into the bird clade and is most similar to goose TRIM32 (up to 98.3%), and shares 84.9% similarity with mammalian TRIM32. Similar to human TRIM32, duTRIM32 was also found to contain a conserved RING-finger domain, a BBOX domain and a coiled-coil domain, which suggested that duTRIM32 might possess the same functions of mammalian TRIM32.

Recent studies have demonstrated that mammalian TRIM32 is able to positively regulate the type I IFN pathway and cellular antiviral response. Overexpression of mammalian TRIM32 could activate the IFN-β promoter and upregulate the mRNA expression of virus-triggered IFN-β, so as to inhibit the replication of VSV in infected cells (11). In our study, we found that overexpression of duTRIM32 significantly activates the IFN-β promoter and upregulates the expression of related genes in the type I IFN pathway (e.g., IFN-β, IRF7, and Mx). Therefore, our results suggested that duTRIM32 was involved in the activation of the type I IFN pathway.

Reports indicate that the mammalian STING-mediated type I IFN pathway is regulated by several TRIM proteins. Mammalian TRIM32 could target STING for K63-linked ubiquitination and was found to contribute to STING mediated expression of IFN-β (11). However, TRIM29, and TRIM30a were reported to induce K48-linked ubiquitination of STING, thereby degrading STING and inhibiting the innate immune response (19, 20). A recent study showed that duSTING is involved in the induction of IFN-β and inhibits the replication of the duck plague virus *in vitro* (21). In addition, overexpression of duSTING was also demonstrated to inhibit the replication of H9N2 AIV (22). In this study, we found that duTRIM32 directly interacts with duSTING and contributes to the production of IFN-β mediated by duSTING, though the increase level is <2-folds. Furthermore, our results showed that duTRIM32 was demonstrated to ubiquitinate duSTING in 293T cells. Our results suggest that duTRIM32 can positively regulate duSTING mediated by the type I IFN pathway by directly interacting with duSTING.

Several mammalian TRIM proteins are reported to be involved in host innate immune responses to influenza A virus infection. Overexpression of TRIM19 (PML) may inhibit the replication of the influenza virus (34). Both TRIM22 and TRIM41 have been found to inhibit the replication of influenza A virus by degrading NP proteins (35, 36). TRIM32 was also reported as a restriction factor for influenza A viral infection (12). In this study, we found that the expression of duTRIM32 was significantly upregulated in the lungs and spleens of the H5N6 HPAIV infected ducks at 3 DPI, which suggests that duTRIM32 might be involved in the duck immune response to H5N6 HPAIV infection. Overexpression of duTRIM32 could inhibit the replication of H5N6 HPAIV in infected cells. However, the inhibitory effect of duTRIM32 on H5N6 HPAIV is moderate and not complete. These results reveal that duTRIM32 may also act as an antiviral factor to H5N6 HPAIV infection.

Taken together, we have found that duTRIM32 can positively regulate duSTING mediated type I IFN pathway by directly interacting with duSTING. DuTRIM32 was found to exhibit an antiviral effect against H5N6 HPAIV infection. This helps to delineate the function of duck TRIM proteins in regulating the type I IFN pathway and their antiviral effect on AIV.

## Data Availability Statement

The datasets generated for this study are available on request to the corresponding author.

## Ethics Statement

The animal study was reviewed and approved by the Experimental Animal Administration and Ethics Committee of the South China Agricultural University.

## Author Contributions

SW and PJ designed this study, performed the experiments, and drafted the manuscript. JZ, QX, JL, BH, ZH, JH, SZ, ZC, and BZ assisted with animal experiment. ML participated in writing the discussion. All authors have read and approved the final manuscript.

### Conflict of Interest

The authors declare that the research was conducted in the absence of any commercial or financial relationships that could be construed as a potential conflict of interest.
