# Supplementary material for: Duck TRIM32 Functions in IFN-β Signaling Against the Infection of H5N6 Highly Pathogenic Avian Influenza Virus
Source: Front Immunol. 2020 Feb 28;11:377. doi: 10.3389/fimmu.2020.00377 (PMC7058987; doi:10.3389/fimmu.2020.00377)

Supplementary materials for figure 8.

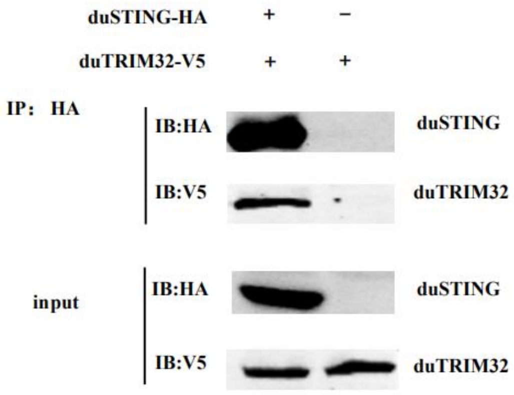

Figure 8. Identification of the interaction between duTRIM32 and duSTING by Co-IP assays. The 293T cells were transfected with the pCAGGS-duTRIM32-V5 and/or pCAGGS-duSTING-HA. After 24 hours, the lysates were immunoprecipitated with anti-HA beads, followed by immunoblotting analysis with the indicated Abs.

Supplementary materials for figure 8.

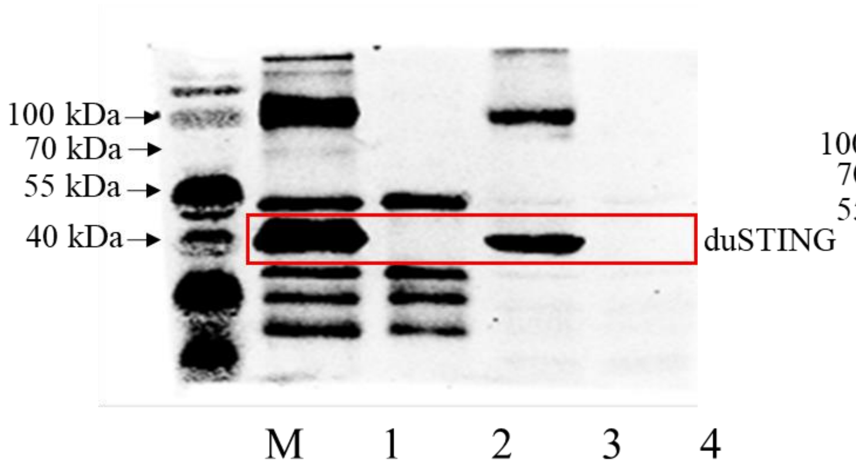

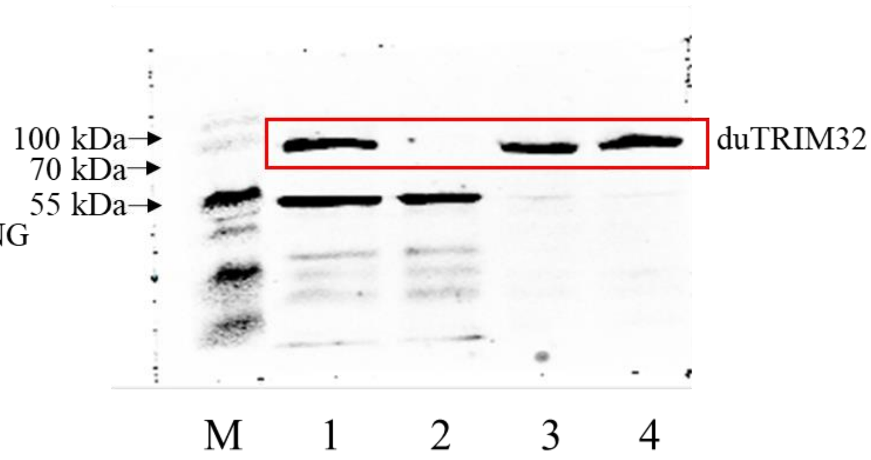

Supplement: Supplementary file 1 [file Data_Sheet_1.PDF]
